# Power and vulnerability: managing sensitive language in organizational communication

**DOI:** 10.3389/fpsyg.2023.1266425

**Published:** 2024-02-23

**Authors:** Patrick G. T. Healey, Prashant Khare, Ignacio Castro, Gareth Tyson, Mladen Karan, Ravi Shekhar, Stephen McQuistin, Colin Perkins, Matthew Purver

**Affiliations:** ^1^School of Electronic Engineering and Computer Science, Queen Mary University of London, London, United Kingdom; ^2^Department of Electronic and Computer Engineering, Hong Kong University of Science and Technology, Hong Kong, China; ^3^School of Computer Science and Electronic Engineering, University of Essex, Colchester, United Kingdom; ^4^School of Computing Science, University of Glasgow, Glasgow, United Kingdom; ^5^School of Computer Science, University of St Andrews, St Andrews, United Kingdom; ^6^Jožef Stefan Institute, Ljubljana, Slovenia

**Keywords:** politeness, communication, dialogue, power, organization

## Abstract

Organizational responsibilities can give people power but also expose them to scrutiny. This tension leads to divergent predictions about the use of potentially sensitive language: power might license it, while exposure might inhibit it. Analysis of peoples' language use in a large corpus of organizational emails using standardized Linguistic Inquiry and Word Count (LIWC) measures shows a systematic difference in the use of words with potentially sensitive (ethnic, religious, or political) connotations. People in positions of relative power are ~3 times less likely to use sensitive words than people more junior to them. The tendency to avoid potentially sensitive language appears to be independent of whether other people are using sensitive language in the same email exchanges, and also independent of whether these words are used in a sensitive context. These results challenge a stereotype about language use and the exercise of power. They suggest that, in at least some circumstances, the exposure and accountability associated with organizational responsibilities are a more significant influence on how people communicate than social power.

## 1 Introduction

There is a belief that people in positions of power can speak more freely than their juniors because they are less vulnerable to criticism. This belief has informed work on politeness in conversation, with the use of impolite language sometimes identified with the exercise of social power (see e.g., Bousfield and Locher, 2008; Danescu-Niculescu-Mizil et al., 2012; Gilbert, 2012; Wang, 2021; Paik and van Swol, 2022). There is also a contrary belief: people in positions of power are monitored and held to account in ways that junior people are not, making them more cautious about what they say and how they say it. We explore these conflicting beliefs in the context of a large corpus of email communication in an international standards organization. The results suggest that, in at least some contexts, vulnerability is a more significant influence on language use than relative social power.

Brown and Levinson's (1987) landmark study of politeness observed that sensitive topics, such as “politics, race, religion, women's liberation” (p. 314), create a social risk for both speakers and hearers. These topics are sensitive because they touch on personal and group identities that are often implicated in interpersonal and group conflict (Triandis, 2000; Levy et al., 2022). In Brown and Levinson's (1987) politeness theory, raising these topics is a potentially divisive, face threatening act because it can suggest that the speaker is insensitive to the hearer's desire to have their social identity or positive face respected. Similarly, these topics can pose a threat to the self-image of a speaker if, for example, a hearer publicly challenges the appropriateness of their remarks.

Judgements about what kinds of talk are (in)appropriate are complex and vary between times, contexts, individuals, and speech communities. Moreover, people can sometimes introduce sensitive topics with the intention of strengthening a relationship, e.g., as a form of self-disclosure or demonstration of informality or community solidarity, just as much as with the intention of threatening a relationship (see e.g., Schnurr et al., 2008).

A significant complicating factor in judgements of what kinds of language are appropriate in a particular situation is social power, defined by Brown and Levinson as mutually agreed relative status. Differences in social power distort the equilibrium of “mutual vulnerability” to face threats (Brown and Levinson, 1987, p. 320). What is the direction of these effects? Brown and Levinson introduce a Power Hypothesis. Roughly, the more powerful a person is, the less vulnerable they are to threats to their own self-esteem (positive face) and the more able they are to resist requests or instructions that might restrict their own freedom of action (negative face). Conversely, they are also less concerned with attending to the face needs of others and, by definition, they are in a position to restrict other people's freedom of action. The Power Hypothesis has informed most of the subsequent quantitative work on politeness and social hierarchies.

An alternative intuition, which we gloss as the Exposure Hypothesis, is that people with higher social status are potentially more vulnerable to face threats. People with significant decision-making responsibilities may also be subject to additional social pressures. They often need to work to build a consensus around decisions and to anticipate and manage potential challenges and objections. Most importantly, they are often also held publicly or legally accountable for those decisions in ways that people without executive power are not. Documents such as emails provide a record that may be scrutinized by a variety of people, in a variety of contexts and over unpredictable timespans. As far as we are aware, the Exposure Hypothesis and the intuition behind it has not previously been systematically tested.

These two hypotheses make contrasting predictions. The Power Hypothesis suggests that people with more social power should be more likely to use potentially sensitive words, whereas the Exposure Hypothesis suggests that they should be less likely.

Previous work testing the relationship between patterns of language use and power in large datasets of institutional communication has found mixed results. Gilbert's (2012) study of the Enron corpus looked at whether differences in language use could distinguish between emails sent up and down the corporate hierarchy. Although some specific phrases predict the direction of the communication in this corpus -e.g., “the ability to,” “attach,” and “I took” associate with emails up the corporate hierarchy and “have you been,” “to manage the,” and “you gave” do not- there is no clear generalization across the two sets of predictor phrases. A follow-up LIWC analysis (see below) looking at several different generic aspects of language use found a difference in the level of evidence of cognitive processes suggesting that junior people may prefer to provide senior people with answers rather than reasoning (Gilbert, 2012). Eckhaus and Sheaffer (2018) analyzed changes in pronoun use over time by two of the most senior managers at Enron and connected a shift toward use of first person singular (“I”) to an increase in managerial hubris over time. Danescu-Niculescu-Mizil et al. (2012) analyzed Wikipedia edit discussions and oral arguments before the US high court. They found evidence that people with lower social power tend to accommodate their language use -defined as matching of function words- more to the language use of those higher in power than vice versa. However, Tan et al. (2016) complicate this picture, finding that lower accommodation -defined here in terms of match/mismatch of content words- appears to make arguments more persuasive in Reddit discussions. Cotterill et al. (2015) provide data from an experimental task in which power is directly controlled using a manipulation of roles. They show that training a classifier on a variety of stylometric features including characters per word, punctuation marks, interjections, polite expressions, and function words, can distinguish between messages up, across, and down the experimentally created hierarchy.

Overall, the picture from previous studies is mixed and, while there is evidence of a systematic relationship between language use and social power, it is not clear exactly what that relationship is. There are several factors contributing to this mixed picture. There is a lack of consensus about what aspects of language use are measured: e.g., function words vs. content words vs. stylometric features; the underlying processes assumed to be associated with social power e.g., accommodation vs. cognitive demands; and about the possible connections between these processes and features of language use. It is also likely that norms about what is and is not appropriate vary across different institutional contexts and cultures and we return to this point in the discussion.

To try to disentangle some of these issues and to test Brown and Levinsons' original predictions, we focus directly on language relating to politics, race, and religion. We analyze a large corpus of organizational email communication between people with different levels of social power operationalized in terms of their organizational role. We use this data to test the contrasting predictions of the Power Hypothesis and the Exposure Hypothesis for the use of potentially sensitive language.

## 2 Methods

The dataset consists of emails produced by the Internet Engineering Task Force (IETF). The IETF is a debate-based, consensus-driven forum that brings together multiple stakeholders (industry, academia, civil society) to agree on many of the key technical standards (e.g., TCP/IP, HTTP) that ensure the Internet works. The IETF is structured into Working Groups (WGs), each with a particular technical focus (e.g., HTTP protocol) and a special mailing list (most IETF works takes place via email). WG chairs facilitate the work with responsibilities including moderating mailing lists, organizing meetings, setting the agenda, and judging consensus on major decisions. We use two publicly available data sources: the IETF mail archives and the Datatracker.^1^ The mail archives cover WG activities, meetings, and administration.^2^ The Datatracker provides information about organizational roles of participants.^3^

The sample consists of all WG email communications in 2019, limited to the last “pre-COVID” year for which we have complete data. We chose this year to avoid the possible impact of the sudden shift to online communication during the pandemic. Following the approach used by McQuistin et al. (2021) and Khare et al. (2022), each email is coded for the WG list in which it occurs and the identity of the sender and then anonymized as a numeric ID with their organizational role. We use only the aggregated data in the statistical analysis below. We distinguish three organizational roles as an index of the relative level of social power someone has in a particular workgroup and its associated email list:

1. **Group chair** codes whether an email is produced by the current chair of the workgroup in which the email occurs.
2. **Other chair** codes whether an email is produced by someone who is the chair of a different workgroup from the one in which the email occurs.
3. **None** codes whether an email originates from people who are not and have not been (at the time of sampling) a workgroup chair.

We order relative social power in these roles in a particular workgroup as Group Chair > Other Chair > None on the basis of their relative influence over group decision making and reporting responsibilities with respect to the workgroup and the IETF.

Three Linguistic Inquiry and Word Count (LIWC-22: Boyd et al., 2022) categories are used to provide an index of potentially sensitive language. We use the pre-defined LIWC measures closest to those cited by Brown and Levinson (see above):

1. **Politics:** words commonly used in political discussions (e.g., congress, parliament, president, democratic) or legal (court, law) discourse.
2. **Ethnicity:** words that identify national, regional, linguistic, ethnic, or racial identities.^4^
3. **Religion:** use of religious words such as “church, altar, god, Christmas, hell, mosque, temple.”^5^

The LIWC categories are standardized measures that have been validated by human judges and explored in a variety of datasets (Boyd et al., 2022). It is important to note that these three LIWC-22 categories encode only mentions of specific words in a category; context is not considered and there is no attempt to disambiguate words that might have alternative senses in this technical domain. This means that, for example, questions about European IP address assignment policies and comments about British humor are both categorized as LIWC Ethnicity. We do not attempt to separate these cases but address this point in the discussion.

## 3 Results

Each email is coded according to the working group it is in and who sent it (Group Chair /Other Chair/None). The emails are pre-processed to remove subject lines, headers, and any embedded quotes from previous emails, yielding 101,857 texts by 2,300 individuals across 176 group mailing lists. Each text is assigned a score on each of the three language categories using the LIWC software and the resulting scores are averaged for each individual sender for statistical analysis.

The LIWC category scores are positively skewed, so Generalized Linear Mixed Model (GLMM) with a Gamma Distribution^6^ and Log link is used for the statistical analysis, but means are reported on the original scale. Organizational Role (Group Chair /Other Chair/None) is included as a fixed factor. Effect sizes for the pairwise comparisons are computed using Hedges g because of unequal sample sizes in the comparisons. The three analyses indicate a consistent pattern across the different LIWC categories.

GLMM analysis of the LIWC Politics measure shows an overall main effect of Role [*F*_(2,790)_ = 20.5, *p* < 0.001]. Pairwise comparisons show that None are reliably more likely than either Other Chair or Group Chair to use political words [Group Chair vs. None: *t*_(790)_ = −6.13, *p* < 0.001, Hedges' g = 0.32; Other Chair vs. None: *t*_(790)_ = −2.83, *p* = 0.010, = Hedges' g = 0.21]. Group Chair and Other Chair are not reliably different [*t*_(790)_ = 1.72, *p* = 0.086].

GLMM analysis of LIWC Ethnicity also shows a main effect of Role [*F*_(2,227)_ = 25.7, *p* < 0.001]. Pairwise comparisons show that None use more Ethnicity-related words than either Other Chair or Group Chair: [Group Chair vs. None: *t*_(227)_ = −3.89, *p* < 0.001, Hedges' g = 0.33; Other Chair vs. None: *t*_(227)_ = −4.478, *p* = 0.010, Hedges' g = 0.39]. Group Chair and Other Chair are not reliably different [*t*_(227)_ = 0.93, *p* = 0.354].

GLMM for LIWC Religion also shows an overall effect of Role [*F*_(2,408)_ = 5.12, *p* = 0.006]. The pairwise comparisons between Group Chair and None [*t*_(408)_ = −2.76, *p* = 0.018, Hedges' g = 1.5] and Other Chair and None [*t*_(408)_ = −2.39, *p* = 0.03, Hedges' g = 0.95] are also reliable.

**Figure 1:**
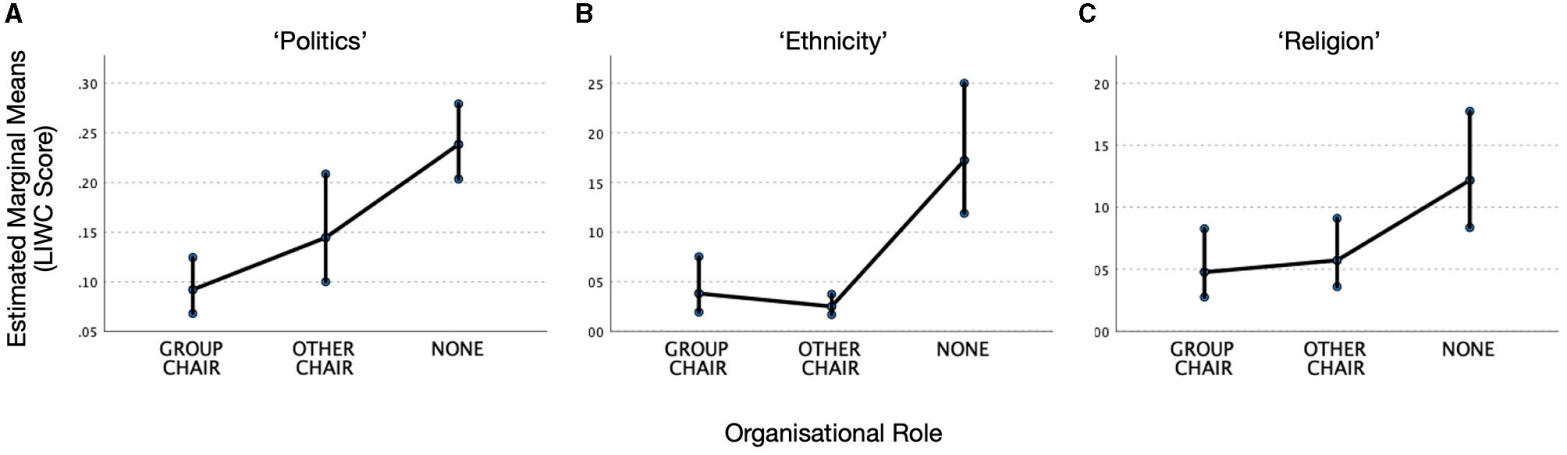
Patterns of language use by organizational role. Estimated marginal means for the LIWC categories: **(A)** Politics, **(B)** Ethnicity, and **(C)** Religion. Error bars show 95% confidence intervals.

The results for all three analyses are illustrated in Figure 1. The consistent pattern is that people with organizational responsibilities (Group Chair or Other Chair) are less likely to use words potentially connected with sensitive topics than those without organizational responsibilities (None), even though they are all part of the same discussions on the same email lists. Averaging across the two Chair categories, the means indicate that people in positions of social power are 3.2 times less likely to use sensitive language overall than those who are not (by category: Politics: 2.0 times; Religion 2.3 times; Ethnicity 5.5 times).

It is worth noting that, in absolute terms, potentially sensitive language use is rare in this dataset, as might be expected for primarily work-related communication. The modal LIWC score in both raw data and the averages scores is zero. For comparison, the reported mean percentage occurrence of each category in the present dataset, natural conversation, and Twitter (now X) is summarized in Table 1 (Boyd et al., 2022). Despite the low overall incidence of potentially sensitive language, the results show systematic differences in language use according to organizational role with medium to large effect sizes.

**Table 1:** Comparison of relative occurrence of words in the LIWC Politics, religion and ethnicity categories in the IETF corpus, and the natural conversation and twitter samples reported by Boyd et al. (2022).

**Dataset:**	**Politics**	**Ethnicity**	**Religion**
Natural conversation	0.10 %	0.16%	0.11%
Twitter (X) sample	0.42%	0.16%	0.53%
IETF email data	0.07%	0.02%	0.01%

Numbers represent mean percentage occurrence in each corpus.

*Post-hoc* Chi^2^ tests were used to assess whether people who use one form of sensitive language are also more likely to use other forms.^7^ The data for each participant and each LIWC category were recoded into binary variables; 0 where the mean instances of a category were 0 and 1 otherwise. The results show reliable associations between each pair of LIWC categories [Politics and Ethnicity: Chi${\;}_{\left(1\right)}^{2}$ = 184, *p* < 0.001, *n* = 2,209; Politics and Religion: Chi${\;}_{\left(1\right)}^{2}$ = 342 *p* < 0.001, *n* = 2,209; Religion and Ethnicity: Chi${\;}_{\left(1\right)}^{2}$ = 403, *p* < 0.001, *n* = 2,209]. Overall, people who use one category of sensitive language are also more likely to use other.

## 4 Discussion

People who have more social power within the IETF, i.e., WG chairs, are systematically less likely to use potentially sensitive words. Given that these interactions all occur on shared mailing lists, this also implies that when WG chairs encounter these words they tend not to reciprocate or align on them. This pattern of results is consistent with the Exposure hypothesis but incompatible with the Power hypothesis.

As noted in the introduction, prior corpus work has typically assumed the basic relationship between power and impoliteness and then sought to identify the linguistic markers that best index that relationship. The analysis reported here also finds a systematic relationship between language and power but tests the direction of this relationship, i.e., whether relative social power is linked to more or less use of potentially sensitive language. The results suggests that, in at least some contexts, the relationship runs in the opposite direction to the one normally assumed: people in positions of power appear to avoid using potentially sensitive words. This finding also runs counter to the more general stereotype of assertive and competitive leaders who use language to project power and confidence, sometimes in overtly hostile ways (see e.g., Tepper, 2007; Koenig et al., 2011; Jordan et al., 2019).

The results have an interesting possible parallel with the language of politicians who also avoid potentially sensitive expressions in exposed, public contexts (see e.g., Bavelas et al., 1990; Obeng, 1997; Bull, 2015). Equivocation by politicians appears to be specifically associated with contexts in which they must contend with the conflicting interests of different groups in building a broader coalition. This description could equally apply to the situations IETF workgroup chairs encounter. However, the LIWC categories studied here relate to social identities and not to conflicting technical or professional interests. It would be interesting to investigate whether sensitivity to potential organizational conflict or sensitivity to wider social norms are more influential in these effects.

Importantly, as noted above, the LIWC categories are not sensitive to linguistic context. So, for example, the use of the word “Christian” as a first name or as a reference to religious belief are not distinguished. One possibility is that this insensitivity to context merely adds noise to a large dataset; however, the effects are still large enough that we observe systematic differences in usage. Another possibility is that the context independence of the LIWC categories accurately reflects a degree of context independence in the way people process potentially sensitive words (see e.g., Madan et al., 2017). Speculatively, people might avoid potentially sensitive language even in apparently unproblematic contexts because alternative meanings are still activated during language production and comprehension (see e.g., Rodd, 2018; Hansen et al., 2019). Something similar may be involved in the removal of, for example, color terms from ostensibly non-racial contexts such as “black cloak” and “white with fear” (see O'Neill, 2023). Exploration of this kind of generalized inhibitory effect would require controlled experiments on processing of LIWC target words in different linguistic and social contexts.

The relationship between language use and social power observed here is likely to depend to some extent on the institutional context and especially on the relative exposure, i.e., accountability and transparency, of a decision-making process. The IETF is committed to a high degree of accountability and transparency, as demonstrated by the way it documents and publishes its organizational communication in a searchable archive. This relatively high level of public exposure may itself lead people to be more cautious in what they say, although this pressure should apply equally to group chairs and group members. The IETF also emphasizes a relatively open, egalitarian, consensus-building culture that may promote a more careful, considered approach to organizational communication than other organizations (although this culture has also been the subject of criticism, see Cath, 2021). An interesting direction for future research may be to explore how organizational practice and openness affects language use, comparing the IETF data with other public communities (e.g., open-source software development communities, public discussion forums) and more closed organizations to explore whether the behavior observed correlates with organizational openness rather than consensus-driven operation.

An important group of alternative, trait-based explanations for the effects reported above is that the kind of people who move into more senior organizational roles are, by disposition, experience, or training, just less likely to use sensitive language. In this case, the observed differences in language use could be due to the kinds of individuals who typically occupy each role rather than the effects of the situation they find themselves in. The data presented here are compatible with both interpretations, although other analyses for different aspects of language use in the same corpus suggest organizational situation may be more important than individual disposition (see also Bavelas et al., 1990; Khare et al., 2023). Future corpus research could explore this issue using longitudinal analysis of the IETF mailing list archives as participants change roles or carry out synchronic comparisons of people in different situations. Whether the working group chairs adjust their language due to the organizational situation they are in or because of the type of person they are, both explanations run counter to the assumption that power should be identified with a tendency to use more sensitive language i.e., with the ability to threaten the “face” of others and to resist face threats by others (Eckhaus and Sheaffer, 2018; Jordan et al., 2019). The analysis of sensitive language use needs to be replicated for other large datasets, especially those that represent different organizational contexts with different forms of accountability. The results suggest that quantitative analyses of the relationship between language use and power need to attend to both what is licensed and what may be inhibited and to how this interacts with situations of use. Future work also needs to allow for the relatively fast pace of changes in norms about (in)appropriateness of specific kinds of language use and changes in both leadership stereotypes and observed leadership practices (Lord et al., 2017).

Quantitative analysis also needs to be complemented by in-depth qualitative work that investigates the contexts of use captured by the LIWC categories and how people perceive and, especially, respond to uses of sensitive language by different people in different organizational contexts (see Bousfield and Locher, 2008). Another interesting avenue for future research would be to explore how people in different organizational roles perceive their relative levels of social power and social exposure.

Although positions of social power are often thought of as positions of strength, the results suggest that, in at least some contexts, they can also be positions of vulnerability because of their heightened exposure and this can be reflected in more cautious, circumspect patterns of language use.

## Data availability statement

Publicly available datasets were analyzed in this study. This data can be found at: https://mailarchive.ietf.org/ and https://datatracker.ietf.org/– administrative database of IETF.

## Ethics statement

Ethical approval was not required for the studies involving humans, because this was a retrospective analysis of publicly available data from the IETF. The studies were conducted in accordance with the local legislation and institutional requirements. People provided written informed consent for their data to be published (publicly) in the IETF archive and the IETF approved the use of this data for the study.

## Author contributions

PH: Conceptualization, Funding acquisition, Methodology, Writing—original draft, Writing—review & editing. PK: Data curation, Methodology, Software, Writing—review & editing. IC: Methodology, Supervision, Writing—review & editing. GT: Funding acquisition, Supervision, Writing—review & editing. MK: Data curation, Methodology, Software, Writing—review & editing. RS: Data curation, Methodology, Software, Writing—review & editing. SM: Data curation, Methodology, Software, Writing—review & editing. CP: Funding acquisition, Supervision, Writing—review & editing. MP: Funding acquisition, Methodology, Supervision, Writing—review & editing.

## Footnotes

^1^ https://mailarchive.ietf.org/

^2^https://datatracker.ietf.org/ – administrative database of IETF.

^3^See both http://www.ietf.org/about/note-well/ and the IETF privacy policy available at http://www.ietf.org/privacy-statement/. IETF leadership confirmed that our work conforms with acceptable use.

^4^Words reflecting racial or ethnic slurs and swearwords are excluded from these categories and included as part of the LIWC swear category.

^5^This list is not exhaustive, but a full definition is not provided in the LIWC documentation, so we take some of this on faith.

^6^As a result, the degrees of freedom vary across the three analyses because Gamma distribution ignores zeros.

^7^We are grateful to one of our anonymous reviewers for suggesting this analysis and the inclusion of comparative estimates of overall incidence of sensitive language use.
